# No Alterations in ACL Injury Risk Factors in Preadolescent Elite Female Handball Players Following an Eight‐Week Targeted Training Intervention: A Randomised Controlled Trial

**DOI:** 10.1155/tsm2/2570210

**Published:** 2026-01-02

**Authors:** Niels J. Nedergaard, Jesper Bencke, Johanna Egholm, August L. Nielsen, Anke N. Karabanov, Jesper Lundbye-Jensen, Mette K. Zebis

**Affiliations:** ^1^ Human Movement Analysis Laboratory, Department of Orthopaedic Surgery, Amager-Hvidovre Hospital, Copenhagen University Hospital, Copenhagen, Denmark, gentoftehospital.dk; ^2^ Department of Nutrition, Exercise and Sports (NEXS), Section for Movement & Neuroscience, University of Copenhagen, Copenhagen, Denmark, ku.dk; ^3^ Department of Midwifery, Physiotherapy, Occupational Therapy and Psychomotor Therapy, Faculty of Health, University College Copenhagen, Copenhagen, Denmark, ucc.dk; ^4^ Institute of Sports Medicine Copenhagen, Bispebjerg and Frederiksberg Hospital, Copenhagen, Denmark, bispebjerghospital.dk

**Keywords:** change-of-direction, injury prevention, knee biomechanics, medial hamstring, neuromuscular training

## Abstract

Adolescent female handball players are at elevated risk of anterior cruciate ligament (ACL) injury. Early interventions during preadolescence seem crucial for improving motor strategies and potentially preventing injuries. This randomised controlled trial on preadolescent female handball players investigated the effects of an 8‐week ACL injury prevention program (ACL‐IPP) targeting modifiable neuromuscular and biomechanical risk factors and assessed retention effects 8 weeks post‐intervention. Thirty‐five elite preadolescent female handball players (11–13 years old) were randomised to the ACL‐IPP group (*n* = 18) or a control group that performed shoulder injury prevention exercises (*n* = 17), with supervised training conducted for 15 min twice weekly. Biomechanical and neuromuscular assessments of participants’ side‐cutting manoeuvres were performed at baseline, immediately after the 8‐week intervention (post‐intervention) and at an 8‐week follow‐up (16 weeks from baseline). The primary outcome was the between‐group change in *m*. semitendinosus EMG preactivity, measured during the 50 ms preceding initial contact of the side‐cutting manoeuvre at 8 weeks. There were no significant between‐group differences in changes in semitendinosus preactivity (*p* = 0.950), isometric hip external rotation strength, hip flexion, knee internal rotation at initial contact or peak knee abduction moments of the handball‐specific side‐cut manoeuvre. Similarly, no retention effects were observed after 16 weeks. These findings suggest that the implemented 8‐week ACL‐IPP was insufficient to elicit meaningful improvements in ACL injury risk factors in this population. Greater training volume, different exercise selection and/or extended intervention duration may be required to induce neuromuscular and biomechanical adaptations in preadolescent female handball players.

**Trial Registration:** ClinicalTrials.gov identifier: NCT05955599

## 1. Introduction

Noncontact anterior cruciate ligament (ACL) injuries remain a significant concern in female team sports [1, 2], including handball [3], with adolescent females showing the highest incidence rates [3, 4]. These injuries have serious consequences, leading to long rehabilitation periods or permanent cessation of sports participation, increased risk of secondary ACL ruptures, particularly in adolescent athletes [5, 6], elevated risk of early‐onset osteoarthritis [7] and diminished quality of life [8].

Similar to other team sports, most noncontact ACL injuries in handball occur during side‐cutting and single‐leg landing [9, 10], where high levels of neuromuscular strength and motor control are needed to counteract the external forces that elevate the load on the knee and ACL [11]. Neuromuscular exercise programs incorporating balance, strength and landing training have shown promising results in reducing injury incidence among adult [12, 13] and adolescent [12, 14, 15] female handball players. However, designing ACL injury prevention programs (ACL‐IPP) tailored to improve known risk factors may improve their effectiveness [16].

In a recent prospective study of 90 adolescent elite female handball and soccer players, we identified four modifiable risk factors for first‐time ACL injury during side‐cutting: low preactivation of the semitendinosus muscle (ST), reduced isometric hip external rotation strength, decreased hip flexion at initial contact (IC) and increased knee internal rotation at IC [17]. Additionally, our data suggest that the peak knee abduction moment, previously identified as a risk factor during bilateral drop jumps [18] and associated with increased injury risk during side‐cutting [9, 10], should be recognised as an ACL injury risk factor. However, the feasibility and effects of prevention programs designed to improve these five specific risk factors in female handball players remain unexplored.

With ACL injury rates peaking during mid‐ to late adolescence (14–18 years) [3, 4], and evidence showing landing mechanics associated with increased ACL injury risk rising during adolescent growth [19, 20], integrating ACL‐IPP in early adolescence might be more effective [21]. Partly because early intervention effects would be preferable in themselves, and additionally since recent studies have found age‐related differences not only in the mechanisms of motor control [22, 23] but also in the effects of motor practice between preadolescents, adolescents and young adults [23]. Based on this, we conducted a randomised controlled trial among elite preadolescent female handball players to investigate the effects of an 8‐week supervised ACL‐IPP designed to improve the modifiable ACL injury risk factors identified by Zebis et al. [17]. Based on a previous case study [24], we hypothesised that kettlebell swing training would increase ST preactivation. Since preventive programs often discontinue after study interventions [25] or are only implemented during pre‐ or post‐season training, our secondary aim was to examine whether players retained neuromuscular adaptations, strength and biomechanical adaptations 8 weeks after completing our ACL‐IPP.

## 2. Materials and Methods

### 2.1. Study Design

We performed an unblinded randomised controlled trial with parallel intervention groups and balanced randomisation. The study was approved by the local ethics committee in the Capital Region of Denmark (H‐21030337). The trial was conducted in close cooperation with amateur handball teams, and the teams’ seasonal competition schedule imposed strict time constraints, requiring the study to begin earlier than originally planned. Consequently, registration occurred retrospectively after the first participants were enrolled. Reporting was conducted in line with the Consolidated Standards of Reporting Trials (CONSORT) 2025 statement for parallel‐group randomised trials [26] (Table S1, Supporting Information 1) and the Consensus on Exercise Reporting Template (CERT) [27] guidelines (Table S2, Supporting Information 2).

### 2.2. Randomisation and Blinding

After the baseline assessments, participants were randomly assigned to either the ACL‐IPP or CON group using blocked balanced randomisation (1:1) within each handball team. The first author conducted the randomisation using a purpose‐built random number generator in Matlab (version R2019a, The MathWorks, Inc., Massachusetts, USA). The first author was unblinded to group allocation to accommodate ethical and practical constraints associated with working in applied youth sports settings, facilitate participant familiarisation with test personnel and ensure safe exercise execution and adjustments. Similarly, blinding of instructors and participants was not feasible. However, the primary and secondary outcomes were collected using automated data processing pipelines, thus minimising the risk of bias due to the lack of assessor blinding. Furthermore, all other members of the research team, including the statistician assisting the data analysis, remained blinded to the group allocation.

### 2.3. Sample Size

We based our sample size calculation (two‐tailed *t*‐test) on our previous study on female athletes ST pre‐activation during side‐cutting [28, 29]. We considered a 15%‐point increase as a clinically relevant improvement, with an expected standard deviation of 15% of max EMG. To ensure 80% power with an alpha level of 0.05, 16 participants were required in each group. Therefore, we aimed to recruit 40 participants to account for potential dropouts.

### 2.4. Participants

Between January and August 2022, we invited under‐13 female handball teams from Copenhagen to participate in the study. Teams had to train at least twice a week, with a minimum of 10 players agreeing to participate for team inclusion. The exclusion criteria were participants outside the under‐13 age group (11–13 years old), less than one year of handball experience, previous ACL injuries, previous lower limb surgeries (e.g. ACL reconstructions) and severe lower limb injuries within the last 3 months. Data collection was performed at the Human Movement Analysis Laboratory at Hvidovre Hospital between January 2022 and February 2023 during the first and second halves of the season. All participants and their parents received written and verbal information about the purpose and procedures of the study. Written consent was obtained from the parents prior to baseline testing.

### 2.5. Intervention

The intervention was implemented during the regular handball season in two under‐13 female handball clubs: one between January and April 2022 (spring term) and the other between September and December 2022 (autumn term). Participants randomised to the ACL‐IPP group performed an 8‐week supervised training intervention, 15 min twice a week, targeting modifiable risk factors for ACL injuries [17]. The program included 3 tracks, each lasting 5 min, with one or two exercises. A track targeting ST preactivation using the kettlebell swing exercise, as described by Zebis et al. [24], and previously reported to be the most effective for ST activation [30, 31]. In the first two weeks, Romanian deadlift (1–2 sets of 10 repetitions) was used to introduce the desired kettlebell swing technique. From week 3 to 8, participants performed 3 sets of 10–15 repetitions of kettlebell swings. Individual kettlebell weights were defined at the first test session, monitored and adjusted during the intervention, with group progression after 4 weeks (average weight from week 1–4: 0.28 ± 0.04 kg/BW, week 5–8: 0.34 ± 0.05 kg/BW). Trained instructors monitored weight adjustment, ensuring that the added weight did not compromise the technique [24]. The second track focused on improving muscle strength through the Nordic Hamstring exercise (1 set of 6–10 repetitions, progressed over eight weeks) and resisted bilateral hip external rotation with adjusted elastic bands (2 sets of 10 repetitions). The third track included neuromuscular handball‐related landing and side‐cutting tasks, focussing on soft landings and alignment of the hip, knee and foot. Each session included two neuromuscular exercises, with 2 sets of 5–10 repetitions per leg. A detailed description of the 8‐week ACL‐IPP and exercise program is provided in Table S3, Supporting Information 3.

CON group participants completed an 8‐week supervised shoulder IPP, 15 min twice a week, addressing the risk factors for overuse shoulder injuries [32]. The program mirrored the ACL‐IPP structure with three training tracks (each lasting 5 min and 1–2 exercises per track). The tracks focused on (1) scapular stability, (2) shoulder external rotation strength and (3) trunk stability and rotational control. Sets (1–3) and repetitions (8–15) varied per exercise and increased progressively.

Prior to the study, all participants regularly performed body‐weight strength exercises two to three times per week as part of their usual handball practice. Both the ACL‐IPP and CON interventions were conducted in groups of 5–11 participants and supervised by 1–2 trained instructors (trained physiotherapy or sports science students) who provided corrections and feedback on exercise quality. The sessions were conducted either before or after regular handball practice. In the minority of cases where sessions preceded regular practice, the initial sets were performed at a submaximal intensity to serve as a preparatory warm‐up. Adherence was predefined as completion of ≥ 75% of the prescribed training sessions. A training session was considered complete when all sets and repetitions of the prescribed exercises were performed at the intended load and with correct technique. Instructors recorded and verified session completion after each session. Participants completing < 75% of prescribed sessions were classified as nonadherent. Additionally, attendance and adverse events were monitored using weekly SMS messages, sent every Sunday throughout the intervention and retention periods. During the intervention, the participants also received verbal reminders at the first session of each week. Adverse events were followed up via nonsystematic telephone interviews. During retention (week 8–16), participants trained normally but were instructed to avoid additional hamstring, hip external rotator strength, or neuromuscular training outside normal practice.

### 2.6. Outcome Measures

The primary outcome was the between‐group difference in the change in ST preactivation of a standardised handball‐specific side‐cut from baseline to the 8‐week follow‐up. ST preactivation was measured with surface electromyography, expressed as the average EMG activity of the 50 ms prior to initial contact (IC) and normalised to the maximal EMG amplitude of an isometric maximal voluntary hamstring contraction (% of max EMG) [17, 28, 29].

Secondary outcomes were between‐group differences in changes in (1) maximal isometric hip external rotation strength, measured with a handheld dynamometer and expressed as N/kg BW; (2) hip flexion; (3) knee internal rotation angle at IC measured with three‐dimensional motion capture and expressed in degrees (°); and (4) peak knee abduction moment within the first 100 ms after IC, recorded from inverse dynamics, expressed as Nm/kg BW from baseline to 8 week follow‐up.

Additionally, between‐group differences in changes from 8‐ to 16‐week follow‐up in ST preactivation, maximal isometric hip external rotation strength, hip flexion angle at IC, knee internal rotation angle at IC and peak knee abduction moment were analysed to explore potential differences after 8 weeks of retention.

### 2.7. Biomechanical Test Procedures

The first author performed all biomechanical tests in the motion capture laboratory at Hvidovre Hospital, Denmark. Following a 10 min standardised warm‐up, which included a series of running, jumping and cutting activities of progressively increasing intensity, participants performed a standardised handball‐specific side‐cut manoeuvre [17]. Participants started at an individual distance (∼4–5 m) in front of a force plate, and side‐cut was performed on their dominant leg towards their preferred throwing arm [33]. The participants were instructed to perform the side‐cut as fast as possible to ensure game‐like intensity. Five successful trials (with the foot within the force plate) were completed in each test session.

Lower‐limb kinematics were recorded at 200 Hz using an eight‐camera Vicon system (T40 cameras, Vicon, Oxford, England) with a CGM2.3 marker set [34] including two additional pelvis markers on the iliac crest to improve pelvis tracking. The ground reaction forces were recorded at 1000 Hz using an AMTI force plate (OR6‐7, AMTI, Massachusetts, USA). The marker trajectory and ground reaction force data were filtered with a zero‐lag fourth‐order low‐pass Butterworth filter with cutoff frequencies of 20 and 50 Hz, respectively. IC was identified when the vertical ground reaction force exceeded a 10‐N threshold. Hip flexion at IC, knee internal rotation at IC and external peak knee abduction moments within 100 ms after IC were calculated from inverse kinematics and kinetics using pyCGM2 [34] integrated functions in Vicon Nexus (version 2.15; Vicon, Oxford, England). Subsequently, the knee internal rotation angle was adjusted according to the participants’ tibial torsion recorded in a static trial. The biomechanical variables have previously demonstrated good between‐session reliability for side‐cutting in elite female handball players [35].

ST EMG was recorded at 1000 Hz using wireless EMG sensors (Cometa, Bareggio, Italy), with bipolar electrodes with a 2.0 interelectrode distance. Raw EMG signals were filtered using a 10‐Hz fourth‐order high‐pass Butterworth filter, smoothed with a moving root‐mean‐square filter (30‐ms window, 29‐ms overlap) and subsequently normalised to maximal isometric voluntary contraction EMG amplitudes, all as previously described [28, 29]. Mean ST preactivation was calculated 50 ms before IC. This approach has shown highly reproducible EMG patterns across repeated side‐cutting trials in female adolescent handball players [28].

Maximal isometric hip external rotation strength of the dominant leg was measured with a handheld dynamometer (microFET2, Hoggan Scientific, Salt Lake City, USA) with participants seated, hip and knee flexed at 90°, as previously described [17], and demonstrated high test–retest reliability [36]. The participants completed a minimum of three trials each. Only the best trial was used for analysis.

To evaluate physical maturity, the participants were asked to complete a diagrammatic version of the Tanner scale [37], with parents or guardians providing assistance as needed.

### 2.8. Statistical Analysis

All outcome variables were analysed by intention‐to‐treat principle. Missing data were replaced by mean group changes for the given time‐period. We used ANCOVA to analyse the ACL‐IPPs effect on changes in outcome measures with group allocation as fixed factor and outcome measure baseline values as covariates. A similar approach was used to explore retention effects, with 8‐week follow‐up values as covariates. We used a one‐sample *t*‐test to test the within‐group effects of the ACL‐IPP (baseline to 8‐week follow‐up) and retention (8‐ to 16‐week follow‐up). Outcome measures were checked for normality by Q–Q plots and Shapiro–Wilk test, and variance homogeneity with Levene’s test. Statistical tests were performed using an α‐level of 0.05 in IBM SPSS Statistics version 29 (IBM Corporation, Armonk, USA).

### 2.9. Deviations From Clinical Trial Report

Our clinical trial registration also includes an age‐related comparison between preadolescent and adult (≥ 18 years) female handball players, which is not reported here. Similarly, the effects of ACL‐IPPs on cortical and muscular activity and visuomotor skill learning in different age groups will be published elsewhere.

## 3. Results

### 3.1. Participants

**Figure 1:**
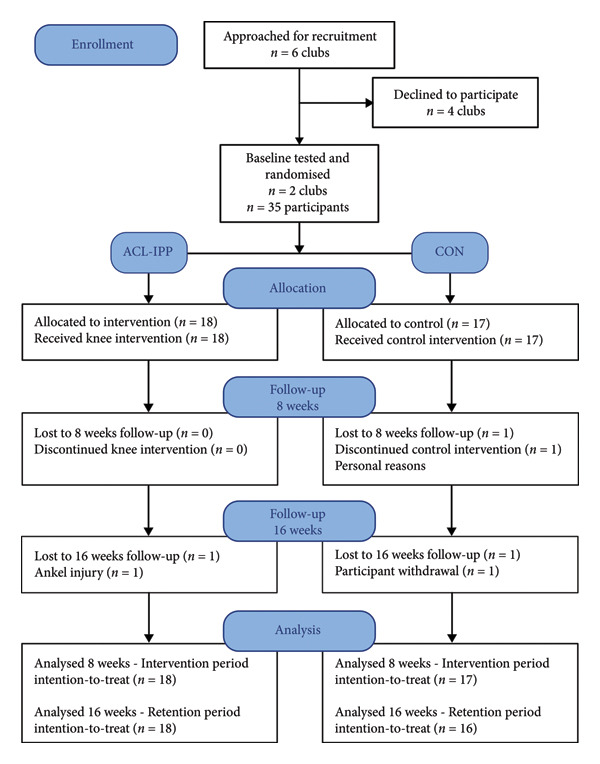
Study flow diagram of enrolment, randomisation and number of participants tested at 8 weeks and 16 weeks, respectively. ACL‐IPP: ACL injury prevention program group. CON: control group, allocated to shoulder injury prevention training.

Six handball teams with eligible under‐13 female players were approached between January and September 2022 (Figure 1). Two clubs agreed to participate, and 35 participants were randomised to either the ACL‐IPP group (*n* = 18) or the CON group (*n* = 17). Similar Tanner stage distributions were observed between groups at baseline; overall baseline characteristics are presented in Table 1. One participant in the CON group quit handball during the 8‐week intervention. Two participants were lost to the 16‐week follow‐up: one from the ACL‐IPP group due to an ankle sprain and one from the CON group who did not attend the follow‐up. The ACL‐IPP group had an average training adherence of 88.5 ± 12.4%, with 16 participants attending at least 16 sessions of training. The CON group had an average training adherence of 85.5 ± 12.9%, with 15 participants attending 16 sessions.

**Table 1:** Baseline characteristics (mean ± SD) of the participants included in the intention‐to‐treat analysis.

	ACL‐IPP (*n* = 18)	CON (*n* = 17)
Age (years)	12.5 ± 0.6	12.6 ± 0.5
Tanner stage (*N*)		
Stage 1	1	0
Stage 2	3	3
Stage 3	10	11
Stage 4	3	3
Stage 5	1	0
Body mass (kg)	52.7 ± 9.8	54.3 ± 7.7
Height (cm)	161.8 ± 16.3	162.6 ± 6.1
Handball experience (years)	5.9 ± 2.1	7.0 ± 1.8
*Primary outcome*		
Semitendinosus preactivation (% of max EMG)	28.7 ± 10.6	35.0 ± 13.3
*Secondary outcomes*		
Hip external rotation MVC (N/kg BW)	2.2 ± 0.3	2.0 ± 0.2
Hip flexion angle at IC (°)	48.3 ± 9.3	45.3 ± 7.1
Knee internal rotation angle at IC (°)	−3.8 ± 5.9	−1.6 ± 8.8
Knee adduction moment (nm/kg BW)	−0.76 ± 0.48	−0.95 ± 0.39

Abbreviations: IC, initial contact; MVC, maximal isometric voluntary contraction.

### 3.2. Primary Outcome

The ACL‐IPP had no effect on change in ST preactivation from baseline to 8‐week follow‐up (Figure 2(a)). The ANCOVA analysis showed no statistical between‐group difference (Table 2) in change in ST preactivation (mean difference 0.3% of max EMG, favouring ACL‐IPP group (CI: −9.2–9.7), *p* = 0.950).

Figure 2Group means ± SD for the ACL injury risk factors at baseline, after the 8‐week training intervention (8 weeks), and after the 8‐week retention period (16 weeks). (a) ST preactivation EMG, (b) hip external rotation strength, (c) hip flexion angle at initial contact, (d) knee internal rotation angle at initial contact and (e) external knee adduction moment within the first 100 ms after initial contact. Boxplots represent the interquartile range and median; shaded areas show distribution density. Individual participants’ mean values at each time point are shown as small circles and connected by thin grey lines.

**Figure:**
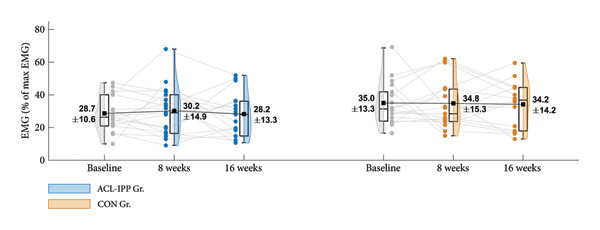
(a)

**Figure:**
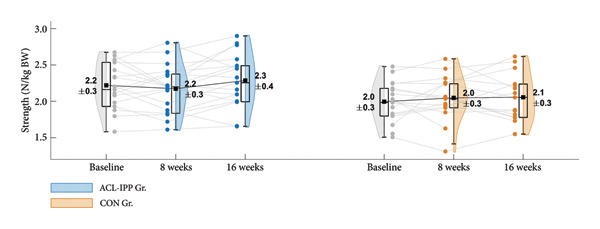
(b)

**Figure:**
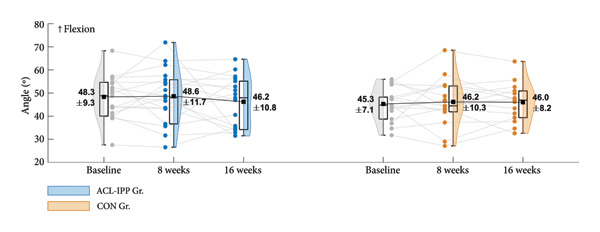
(c)

**Figure:**
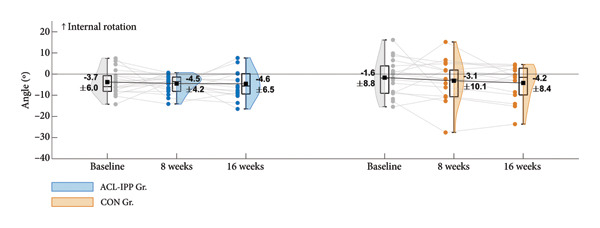
(d)

**Figure:**
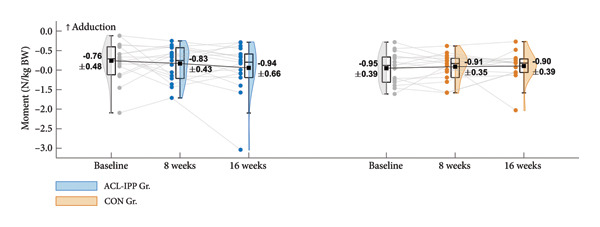
(e)

**Table 2:** Adjusted between‐group mean differences (95% confidence intervals) and ANCOVA statistics.

	Baseline to 8 week		8–16 week	
	Mean difference (95% CI)	*p*	Mean difference (95% CI)	*p*
Semitendinosus preactivation (% of max EMG)	0.3 (−9.2–9.7)	0.950	−3.1 (−11.6–5.5)	0.472
Hip external rotation MVC (N/kg BW)	−0.1 (−0.2–0.1)	0.366	0.2 (0.0–0.3)	0.069
Hip flexion angle at IC (°)	0.1 (−6.0–6.2)	0.980	−1.2 (−5.0–2.3)	0.502
Knee internal rotation angle at IC (°)	0.4 (−4.0–4.7)	0.862	−1.1 (−2.7–5.0)	0.562
Knee adduction moment (nm/kg BW)	0.02 (−0.22–0.26)	0.884	−0.10 (−0.41–0.21)	0.434

Abbreviations: IC, initial contact; MVC, maximal isometric voluntary contraction.

### 3.3. Secondary Outcomes

Similar patterns were found for secondary strength and biomechanical outcomes (Figures 2(b), 2(c), 2(d) and 2(e)), with no significant between‐group differences in changes from baseline to the 8‐week follow‐up (Table 2). No significant between‐group difference in change in ACL injury risk factors were observed after retention (8‐week to 16‐week follow‐up). Finally, no significant within‐group differences were observed for any of the outcomes (Table 3). See Table S4, Supporting Information 4, for un‐normalised ST EMG and hip external rotation strength values.

**Table 3:** Within‐group differences for the ACL‐IPP and CON groups, presented as mean differences (95% confidence interval).

	Baseline to 8 week		8–16 week	
	Mean difference (95% CI)	*p*	Mean difference (95% CI)	*p*
*ACL-IPP*				
Semitendinosus preactivation (% of max EMG)	1.6 (−5.3–8.4)	0.632	−2.59 (−9.79–4.61)	0.459
Hip external rotation MVC (N/kg BW)	−0.1 (−0.2–0.1)	0.341	0.11 (−0.01–0.23)	0.068
Hip flexion angle at IC (°)	0.3 (−3.9–4.6)	0.880	−2.39 (−5.43–0.64)	0.115
Knee internal rotation angle at IC (°)	−0.8 (−5.4–3.8)	0.707	1.22 (−2.69–5.14)	0.518
Knee adduction moment (nm/kg BW)	−0.07 (−0.32–0.17)	0.544	−0.09 (−0.31–0.13)	0.390

*CON*				
Semitendinosus preactivation (% of max EMG)	−1.1 (−8.2–6.0)	0.751	−1.9 (−9.4–5.6)	0.593
Hip external rotation MVC (N/kg BW)	0.0 (0.0–0.1)	0.346	0.0 (−0.2–0.1)	0.739
Hip flexion angle at IC (°)	0.8 (−3.8–5.4)	0.712	0.4 (−3.7–2.8)	0.775
Knee internal rotation angle at IC (°)	0.7 (−4.9–6.2)	0.802	−1.1 (−5.6–3.4)	0.607
Knee adduction moment (nm/kg BW)	0.06 (−0.12–0.23)	0.514	−0.02 (−0.27–0.23)	0.854

*Note:* *p* values display one‐sample *t*‐test statistics (*α*‐level at 0.05).

Abbreviations: IC, initial contact; MVC, maximal isometric voluntary contraction.

### 3.4. Adverse Events

No adverse events related to the ACL‐IPP or CON interventions were reported during the study; however, one participant sprained her ankle during normal handball practice during the retention period.

## 4. Discussion

This is the first study to evaluate the effects of an 8‐week ACL‐IPP targeting modifiable ACL injury risk factors during handball‐specific side‐cutting in preadolescent elite female handball players. Our ACL‐IPP did not improve any of the five ACL injury risk factors, nor did it show retention effects 8 weeks after the completion of the program. The multiple risk factors approach, combined with the short duration and volume of exercises, could explain this.

### 4.1. ST Preactivation

Contrary to our hypothesis and the findings of a case study involving an ACL‐reconstructed female football player [24], our 8‐week ACL‐IPP with a dedicated kettlebell swing track did not improve ST preactivation EMG during handball‐specific side‐cutting. While the relative kettlebell weight was comparable between studies, the lower volume of sets and repetitions in our ACL‐IPP may explain the missing effects (Zebis et al., 2017; 10 sessions, 3–5 sets, 20 repetitions) [24]. Additionally, incorporating the Romanian deadlift for familiarisation during the first two weeks to ensure the correct kettle swing technique reduced exercise volume. Therefore, we recommend that future programs align more closely with the within‐session sets and repetitions of the kettlebell swing exercise described in Zebis et al.’s (2017) protocol and consider increasing the total number of training sessions when the Romanian deadlift is used to introduce the kettlebell swing technique. The baseline ST preactivation during side‐cutting in our cohort matched that in Zebis et al.’s case study (23% of max EMG), suggesting that the initial activation levels do not explain the insignificant training effect. In contrast to our findings, Wilderman et al. reported increased ST activation during side‐cutting following a 6‐week agility training program in female basketball players [38]. Their higher training volume (4 × 15 min per week), and the older age of participants (post‐adolescents), may explain their positive effect compared to our ACL‐IPP, despite our inclusion of a track with neuromuscular jump and cutting exercises. Additionally, our data revealed that a substantial proportion of the preadolescent handball players in our cohort displayed ST preactivation levels below 30% of max EMG (Figure 2(a)), a level associated with an increased ACL injury risk [17]. This underscores the need for feasible IPPs in youth handball that specifically target enhanced ST preactivation during high‐risk side‐cutting manoeuvre.

### 4.2. Secondary Outcomes

Despite the crucial role of external hip rotators in controlling frontal plane knee movements during handball‐specific side‐cutting [11, 33] and their association with increased ACL injury risk [17, 39], this is the first ACL‐IPP to include exercises targeting hip external rotator strength. Our intervention, comprising 2 sets of 10 repetitions twice weekly with a gradual increasing elastic band resistance, was insufficient to produce strength gains. Zebis et al. showed that the ACL injury risk decreases by 23% for every 0.1 N/kg BW increase in the maximal external hip rotator strength [17]. Thus, future ACL‐IPP designs should explore exercise types and increase volumes (sets and repetitions) to obtain such gains and, like our approach, incorporate neuromuscular jump/landing exercises that challenge the hip external rotators.

Consistent with the findings of a meta‐analysis on the ability of injury prevention exercises to modify side‐cutting biomechanics [40], our ACL‐IPP did not improve hip flexion angle or knee internal rotation angle at IC, both of which are associated with increased ACL loading [41, 42]. Similarly, our ACL‐IPP failed to reduce peak knee abduction moments during side‐cutting, in fact, nonsignificant increases were observed post intervention. These results align with the study by Thompson et al. of 51 preadolescent female football players who completed a 30‐min ACL‐IPP in their warm‐up twice weekly over 8 weeks [43]. In contrast, a recent study on 19 adolescent elite female handball players found that a 12‐week neuromuscular training program completed weekly reduced peak knee abduction moments during unanticipated side‐cutting [44].

Technical side‐cutting variables, such as approach speed, vertical impact velocity and cutting angle, explain the substantial variance in peak knee abduction moments among female handball players [45, 46]. Therefore, ACL‐IPP, which focuses on individual side‐cutting techniques, may be more effective than classic neuromuscular jump and landing exercises. Notably, recent studies using biofeedback have shown promising results in reducing peak knee abduction moments in adolescent female handball players [47] and preadolescent female football players [48]. Adapting this approach to modify the ACL injury risk factors explored here is intriguing but currently less feasible in amateur handball settings.

Since our ACL‐IPP did not improve the ACL injury risk factors, we could not assess whether improvements from a short preseason ACL‐IPP were maintained in the season when the program ceased. However, prior research suggests that long‐term prevention programs [49] or maintenance programs [50] are necessary to maintain improvements in landing kinematics and kinetics.

### 4.3. Generalisability

This study was conducted on preadolescent elite female handball players to improve known risk factors of first‐time ACL injury prior to mid‐ to late adolescence, when ACL injury rates peak [3, 4]. The nonsignificant effects of our ACL‐IPP may not generalise to other age groups, preadolescents at different maturation stages, nonelite players, or other team sports. Although individual maturation may influence responsiveness to IPP training, no between‐group differences in Tanner stage distribution were observed in our homogeneous cohort of preadolescent female handball players. This suggests that maturation is unlikely to explain the absence of effects on ACL injury risk factors in the present study. Future studies should investigate whether maturation moderates ACL‐IPP effects on injury risk factors in preadolescent and adolescent female athletes. Additionally, our findings may not be generalisable to preventive programs that focus on a single risk factor, in contrast to our multi‐risk‐factor approach. Although exercises in the ACL‐IPP were performed bilaterally, we only examined the players’ preferred push‐off leg. Previous studies on adolescent elite female handball players found no side‐to‐side differences in biomechanical loading characteristics during handball‐specific side‐cutting [33, 35]; thus, we expect the findings to be generalisable to the players’ contralateral leg.

### 4.4. Limitations

We acknowledge that the lack of blinding of the first author, who was involved in data collection, group allocation and data analysis, is a limitation of this study. However, the primary and secondary outcomes were processed through automated data analysis pipelines and the statistics were reviewed with assistance from a blinded statistician, and group allocation was completed prior to analysis of baseline data, thereby minimising the potential for bias arising from the absence of assessor blinding. Another limitation of the present study is the retrospective registration of the trial, necessitated by seasonal scheduling constraints of the participating teams. Moreover, we acknowledge that retrospective registration represents a limitation, as it may reduce overall transparency. Furthermore, exercise contamination between groups is a potential limitation. To minimise this risk, the ACL‐IPP and CON groups conducted their sessions in different rooms whenever feasible. Moreover, instructors adhered strictly to the assigned protocols, and participants were instructed not to share exercise content across groups. Nevertheless, we believe that the inclusion of a control shoulder IPP is an ethical strength of this study, helping to mitigate concerns about providing an IPP to a subgroup within a handball team. Finally, the ACL injury risk factors targeted in our ACL‐IPP are grounded not only in epidemiological evidence but also in the principles of functional anatomy and biomechanical loading of the ACL during dynamic tasks, such as side‐cutting. While we assume that these risk factors are comparable across age groups in female handball players, further research is required to validate this assumption.

## 5. Conclusions

Our findings demonstrate that an 8‐week ACL‐IPP designed to target multiple modifiable ACL injury risk factors was insufficient to elicit measurable improvements in elite preadolescent female handball players. Improving the potentially elevated ACL injury risk profile in this population may require a greater training volume, potentially integrated throughout the competitive season rather than limited to the preseason, or interventions specifically targeting individual risk factors, such as semitendinosus preactivation or hip external rotation strength. Nevertheless, implementing high‐volume or individualised risk factor programs may be challenging within the practical constraints of amateur handball clubs. These findings highlight the need for future research to develop scalable, developmentally appropriate and context‐sensitive ACL injury prevention strategies that can be feasibly integrated into real‐world youth sports.

## Conflicts of Interest

The authors declare no conflicts of interest.

## Funding

This study was funded by a Novo Nordisk Foundation grant to Team Denmark (Grant number NNF.22SA0078293).

## Supporting Information

Additional supporting information can be found online in the Supporting Information section.

## Supporting information


**Supporting Information 1** Supporting Information 1: Consolidated Standards of Reporting Trials (CONSORT) Checklist.

**Supporting Information 2** Supporting Information 2: Consensus on Exercise Reporting Template (CERT).

**Supporting Information 3** Supporting Information 3: Detailed description of the 8‐week ACL injury prevention program.

**Supporting Information 4** Supporting Information 4: Un‐normalised EMG and strength data.

## Data Availability

The data supporting the findings of this study are available upon reasonable request from the corresponding author. The data are not publicly available due to privacy or ethical restrictions.
