# Supplementary material for: No Alterations in ACL Injury Risk Factors in Preadolescent Elite Female Handball Players Following an Eight‐Week Targeted Training Intervention: A Randomised Controlled Trial
Source: Transl Sports Med. 2026 Jan 2;2026:2570210. doi: 10.1155/tsm2/2570210 (PMC12782339; doi:10.1155/tsm2/2570210)
Supplement: Supplementary file 2 — Supporting Information 2 Supporting Information 2: Consensus on Exercise Reporting Template (CERT). [file TSM2-2026-2570210-s003.pdf]

Supplementary Information 2: Consensus on Exercise Reporting Template (CERT)

This supplementary file provides Consensus on Exercise Reporting Template (CERT) for the training intervention presented in the main manuscript entitled *" No alterations in ACL injury risk factors in preadolescent elite female handball players following an eight-week targeted training intervention: A randomised controlled trial."*

Table S2. Consensus on Exercise Reporting Template for the ACL IPP & CON 8-week training program

Item Category	Item No.	Checklist Item	Training Intervention	
			**ACL IPP Gr.**	**CON Gr.**
**WHAT:** materials	1	Type of exercise equipment	Kettlebells, Elastic bands	Elastic bands, Handballs
**WHO:** provider	2	Qualifications, teching/supervising expertise, and/or training of the exercise instructor	Final Year Physiotherapy (BSc) or Sports Science (BSc) Student	Final Year Physiotherapy (BSc) or Sports Science (BSc) Student
**HOW:** delivery	3	Whether exercises are performed individually or in a group	Group	Group
	4	Whether exercises are supervised or unsupervised	All sessions where supervised	All sessions where supervised
	5	Measurement and reporting of adherence to exercise	Weekly SMS Tracking. Instructors registering attendance	Weekly SMS Tracking. Instructors registering attendance
	6	Details of motivation strategies	Feedback and encouragements provided by instructors.<br>End of session competition/game.	Feedback and encouragements provided by instructors.<br>End of session competition/game.
	7	Decision rules for progressing the exercise program	Could perform the exercises without compromising technique.<br><br>For kettlebell swing load was increased by 4kg after 4 weeks. Progressively increased from 1-3 set with new weight without compromising technique.	Could perform the exercises without compromising technique.<br><br>For the elastic band exercise, participants used a harder band or a narrower grip for more resistance if instructors found it too easy.

			For the hip external rotation exercise, instructors added a second or third elastic bands if exercise looked too easy.	
	8	Each exercise is described so that it can be replicated (eg, illustrations, photographs)	Supplementary Information 3	Contact corresponding author for the detailed shoulder injury prevention program
	9	Content of any home program component	Weekly SMS Tracking	Weekly SMS Tracking
	10	Nonexercise components	Weekly SMS Tracking	Weekly SMS Tracking
	11	How adverse events that occur during exercise are documented and managed	Weekly SMS Tracking, followed by structured telephone interviews.	Weekly SMS Tracking, followed by structured telephone interviews.
**WHERE:** location	12	Setting in which exercises are performed	The club's sports facilities	The club's sports facilities
**WHEN, HOW MUCH:** dosage	13	Detailed description of the exercises (eg, sets, repetitions, duration, intensity)	8 weeks<br>2 x 15 minutes<br>Exercise specific number of sets and repetitions, see Supplementary Information 2	8 weeks<br>2 x 15 minutes<br>Exercise specific number of sets (2-3) and repetitions (8-15)
**TAILORING:** what, how	14	Whether exercises are generic ("one size fits all") or tailored to the individual	Generic set of exercises, with individual loads. Generic progression after 4 weeks.	Generic set of exercises, with individual loads. Generic progression after 4 weeks.
	15	Decision rule that determines the starting level for exercise	All participants started at the same time point.	All participants started at the same time point.
**HOW WELL:** planned, actual	16a	Describe how adherence or fidelity to the exercise intervention is assessed/measured.	Weekly SMS Tracking, followed by structured telephone interviews.	Weekly SMS Tracking, followed by structured telephone interviews.

	16b	Whether the exercise intervention is delivered and performed as planned	Identical	Identical
