# Supplementary material for: No Alterations in ACL Injury Risk Factors in Preadolescent Elite Female Handball Players Following an Eight‐Week Targeted Training Intervention: A Randomised Controlled Trial
Source: Transl Sports Med. 2026 Jan 2;2026:2570210. doi: 10.1155/tsm2/2570210 (PMC12782339; doi:10.1155/tsm2/2570210)
Supplement: Supplementary file 3 — Supporting Information 3 Supporting Information 3: Detailed description of the 8‐week ACL injury prevention program. [file TSM2-2026-2570210-s002.pdf]

Supplementary Information 3: Detailed Description of the ACL-injury Prevention Program

This supplementary file provides a detailed description of the ACL-injury prevention program presented in the main manuscript entitled *"No alterations in ACL injury risk factors in preadolescent elite female handball players following an eight-week targeted training intervention: A randomised controlled trial."*

Table S3. Overview of the 8-week ACL-injury prevention program (ACL-IPP), including exercises, sets, repetitions, and loads for individual weeks.

Week	Track	Exercise	Sets	Reps.	Load
**1**	ST-activation	Romanian Deadlift	2	10	KB
		Kettlebell Swing	1	10	KB
	Strength	Nordic Hamstring	1	6	BW
		Bilateral Resisted Hip External Rotation	2	10	EB
	Landing Technique	Run up with bilateral stop-jumps	2	5	BW
		Run up with single-leg diagonal forward jump	2	5	BW
**2**	ST-activation	Romanian Deadlift	1	15	KB
		Kettlebell Swing	2	10	KB
	Strength	Nordic Hamstring	1	6	BW
		Bilateral Resisted Hip External Rotation	2	10	EB
	Landing Technique	Kangaroo Jumps	1	5	BW
		Resisted Kangaroo Jumps	1	5	BW+EB
		Run up with single-leg diagonal forward jump	2	5	BW
**3**	ST-activation	Kettlebell Swing	3	10	KB
	Strength	Nordic Hamstring	1	6	BW
		Bilateral Resisted Hip External Rotation	2	10	EB
	Landing Technique	Run up with single-leg side jump	2	5	BW
		Resisted Kangaroo Jumps	2	5	BW+EB
**4**	ST-activation	Kettlebell Swing	3	10	KB
	Strength	Nordic Hamstring	1	6	BW
		Bilateral Resisted Hip External Rotation	2	10	EB
	Landing Technique	Run up with single-leg side jump	2	5	BW
		Resisted Kangaroo Jumps	2	5	BW+EB
**5**	ST-activation	Kettlebell Swing	3	10	KB
	Strength	Nordic Hamstring	1	8	BW
		Bilateral Resisted Hip External Rotation	2	10	EB
	Landing Technique	Single-leg forward jump with 90 in-air rotation	2	6	BW
		3-step handball sidecut with jump shoot and bilateral landing	2	6	BW
**6**	ST-activation	Kettlebell Swing	3	10	KB
	Strength	Nordic Hamstring	1	8	BW
		Bilateral Resisted Hip External Rotation	2	10	EB
	Landing Technique	Bilateral Jump with in-air push followed by single-leg landing	2	6	BW
		Resisted Kangaroo Jumps with sidejumps	2	9	BW+EB
**7**	ST-activation	Kettlebell Swing	3	15	KB
	Strength	Nordic Hamstring	1	10	BW
		Bilateral Resisted Hip External Rotation	2	10	EB
	Landing Technique	Single-leg forward jump with 90 in-air rotation	2	6	BW
		3-step handball sidecut with jump shoot and bilateral landing	2	6	BW

**8**	ST-activation	Kettlebell Swing	3	15	KB
	Strength	Nordic Hamstring	1	10	BW
		Bilateral Resisted Hip External Rotation	2	10	RB
	Landing	Bilateral Jump with in-air push followed by single-leg landing	2	6	BW
	Technique	Resisted Kangaroo Jumps with sidejumps	2	6	BW

KB: Kettlebell; BW: Body Weight; EB: Elastic Band

Detailed description of the individual ACL-IPP exercises

ROMANIAN DEADLIFT

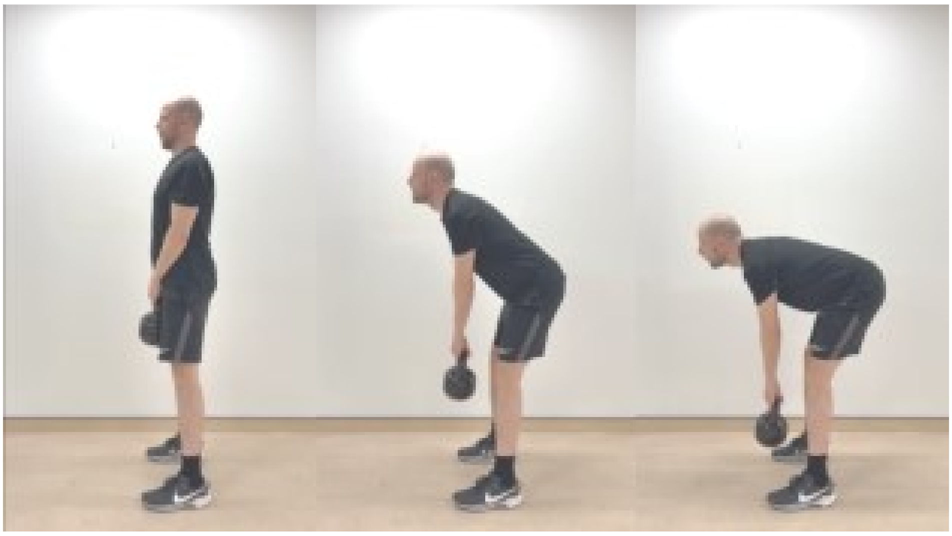

Track: ST-activation

Sets: 1-2

Repetitions: 10-15

Duration: 5 minutes

Load: Individual

The participant stands while holding the kettlebell with both hands, feet parallel and shoulder-width apart. The participant is instructed to flex the hips and push them backward while keeping the upper body straight until the kettlebell reaches just below knee height. The knees should remain slightly flexed (~10–15°) throughout the movement. The kettlebell is then lifted back to the starting position by extending the hips and knees until the participant is standing upright.
The Romanian deadlift exercise was used as a familiarization exercise for the kettlebell swing, with a special focus on maintaining slight knee flexion throughout the movement.

KETTLEBELL SWING

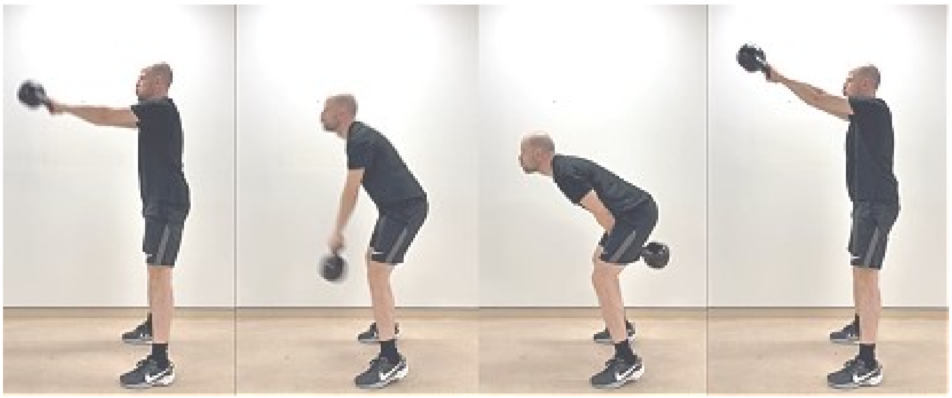

Track: ST-activation

Sets: 1-3 *(3 from week 3)*

Repetitions: 10-15

Duration: 5 minutes

Load: Individual, but without compromising proper technique

The participant stands while holding the kettlebell with both hands, feet parallel and shoulder-width apart, and instructed to maintain a neutral spine throughout the movement. The participant is then instructed to forcefully swing the kettlebell back between the legs by flexing the hips while keeping the knees slightly bent (~10–15°). This is followed by a quick reversal of direction with an explosive hip extension, propelling the kettlebell to chest level. At this point, the hips and knees are fully extended, and the participant is standing upright. EMG analysis has shown that this technique results in high activation levels of the semitendinosus muscles.

NORDIC HAMSTRING

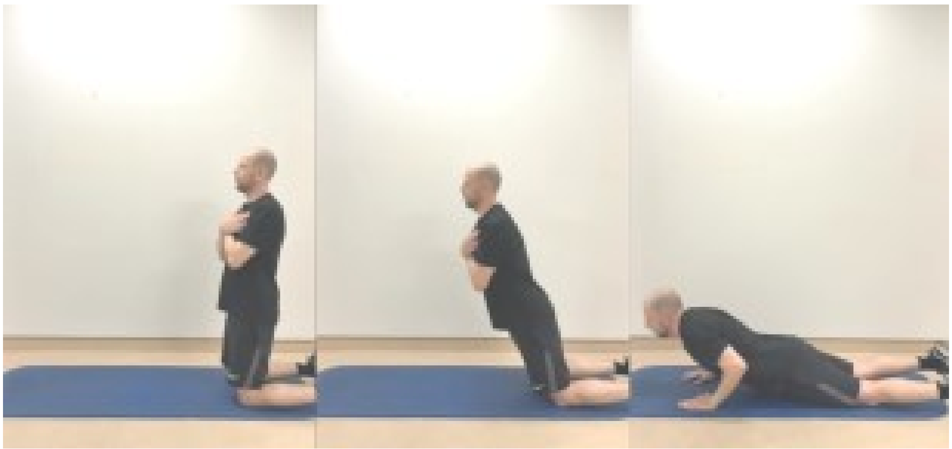

Track: Strength

Sets: 1

Repetitions: 6-10

Duration: 2½-3 minutes

The participant kneels on a soft surface (e.g., a balance mat or gym mat) while a partner holds their ankles. The participant is then instructed to slowly lean the upper body forward while maintaining a neutral spine and pelvis, using the hamstrings to resist falling forward for as long as possible. Use their arms to avoid falling and push-back with both arms to return to the start position.

BILATERAL RESISTED HIP EXTERNAL ROTATION

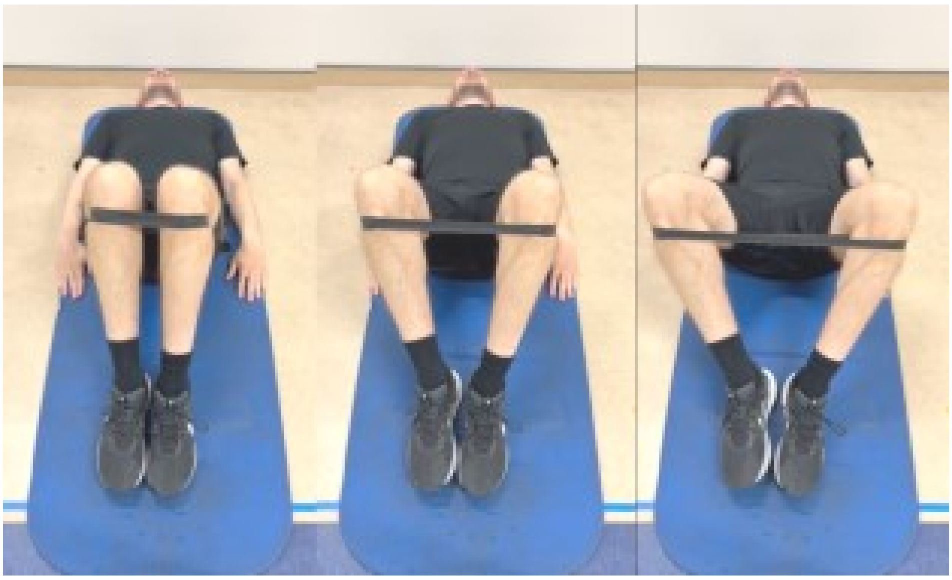

Track: Strength

Sets: 2

Repetitions: 10

Duration: 2½-3 minutes

Load: Individual, but typically between 1-3 elastic bands.

The participant lies supine on the floor, with the elastic band placed proximal to the patella, the hips flexed at ~45°, the knees flexed at ~90°, and the feet on the ground. The participants is instructed to slowly move the knees as far as possible apart, using their hip external rotators, and slowly back to the start position. To ensure a slow and controlled movement, participants were instructed to count to 3 during both the external and internal rotations phase.

RESISTED KANGAROO JUMPS

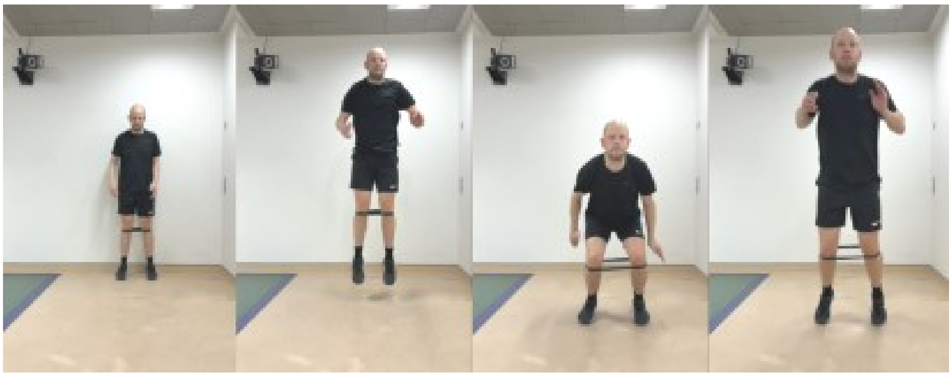

Track: Landing Technique

Sets: 2

Repetitions: 5-6

Duration: 2½-3 minutes

From a standing position with feet shoulder-width apart, participants are instructed to jump forward and land on both legs, lowering their body's center of mass and flexing their knees. They should focus on stabilizing the knees in the frontal plane, avoiding knee valgus, and keeping the knees over the toes. From this position, the participant jumps forward again, continuing until 5–6 consecutive jumps are completed.

Phase 1: The kangaroo jump exercise is performed with external resistance.

Phase 2: An elastic band is placed just above the patella to force the knee into valgus.

Phase 3: A bilateral side-jump is introduced every third jump.

RUN UP WITH BILATERAL STOP-JUMPS

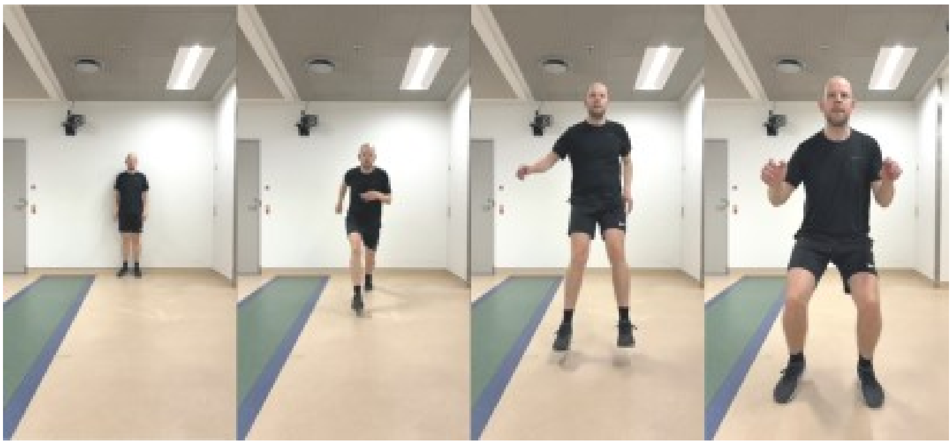

Track: Landing Technique

Sets: 2

Repetitions: 5

Duration: 2½-3 minutes

From a standing position, participants are instructed to run forward at a moderate pace and, on the third step, perform a forward jump followed by a bilateral landing and keep the balance for ~3 seconds. For the landing, participants are instructed to land as quietly as possible while maintaining proper lower limb alignment, ensuring that the knees remain over the toes.

RUN UP WITH SINGLE-LEG DIAGONAL FORWARD JUMP

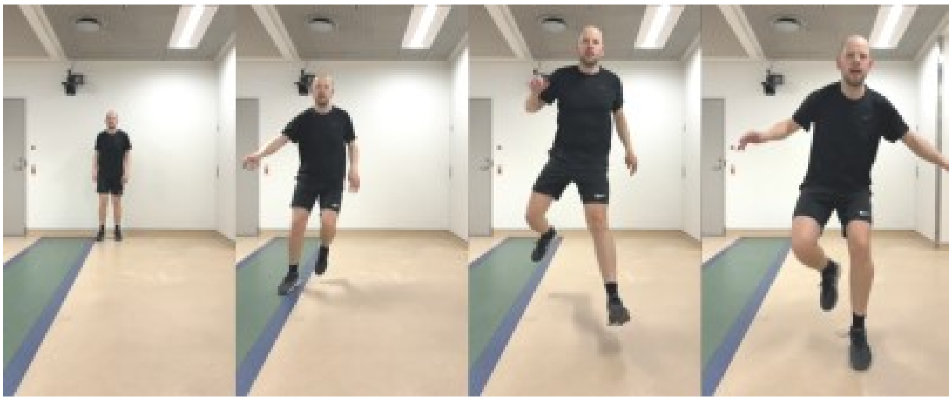

Track: Landing Technique

Sets: 2

Repetitions: 5

Duration: 2½-3 minutes

From a standing position, participants are instructed to run forward at a moderate pace and, on the third step, perform a diagonal forward jump (~45°), landing on the leg opposite to the push-off leg (e.g., pushing off with the left leg and landing on the right leg). Participants were instructed to maintain balance for ~3 seconds, and land as quietly as possible while maintaining proper lower limb alignment, ensuring that the knees remain over the toes.

RUN UP WITH SINGLE-LEG SIDE JUMP

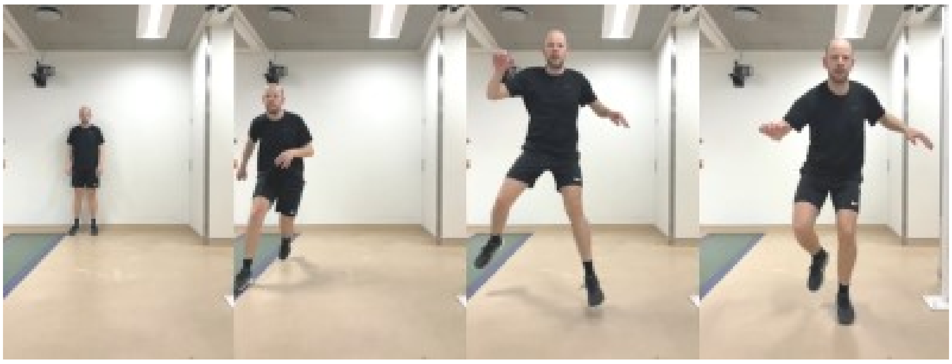

Track: Landing Technique

Sets: 2

Repetitions: 5

Duration: 2½-3 minutes

From a standing position, participants are instructed to run forward at a moderate pace and, on the third step, perform a 45° side-jump, landing on the leg opposite to the push-off leg (e.g., pushing off with the left leg and landing on the right leg). Participants were instructed to maintain balance for ~3 seconds, and land as quietly as possible while maintaining proper lower limb alignment, ensuring that the knees remain over the toes.

SINGLE-LEG FORWARD JUMP WITH 90 IN-AIR ROTATION

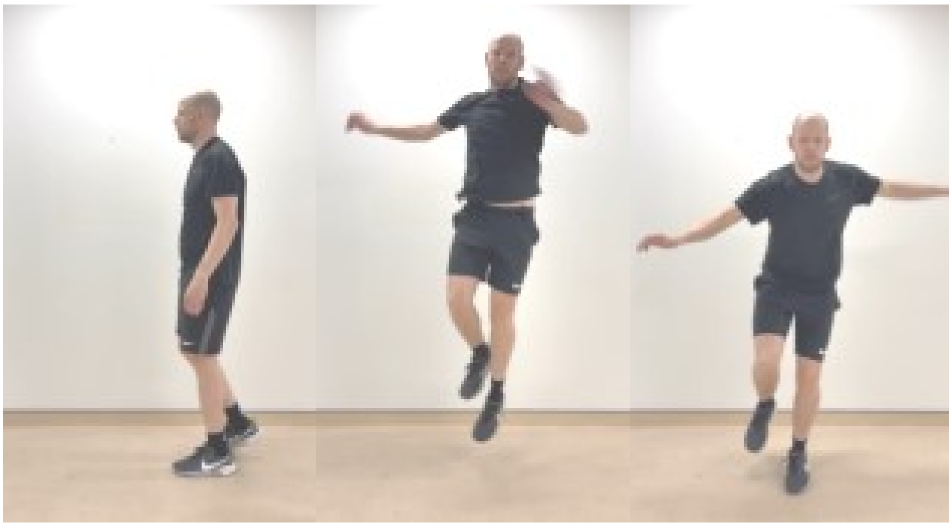

Track: Landing Technique

Sets: 2

Repetitions: 6

Duration: 2½-3 minutes

Participants stand upright on one leg and are instructed to perform an in-air jump with a 90-degree outward rotation, and land on the same leg as the push-off leg. Participants were instructed to focus on proper lower limb alignment in the frontal plane, ensuring that the knee remains over the toes, and maintain balance for ~3 seconds.

3-STEP HANDBALL SIDECUT WITH JUMP SHOOT AND BILATERAL LANDING

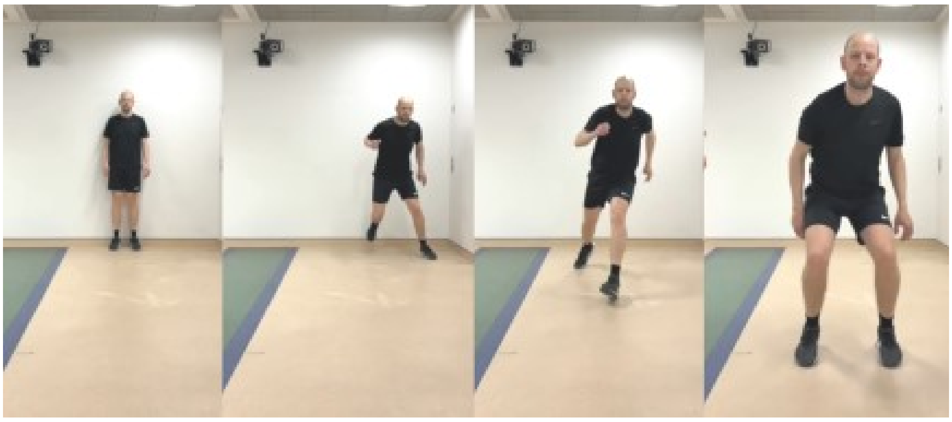

Track: Landing Technique

Sets: 2

Repetitions: 6

Duration: 2½-3 minutes

From a standing position, participants are instructed to perform a three-step handball-specific sidecut with game-like intensity, with a simulated jump shoot on the third step followed by a bilateral landing. Participants were instructed to land as quietly as possible and maintain balance for ~3 seconds.

BILATERAL JUMP WITH IN-AIR PUSH FOLLOWED BY SINGLE-LEG LANDING

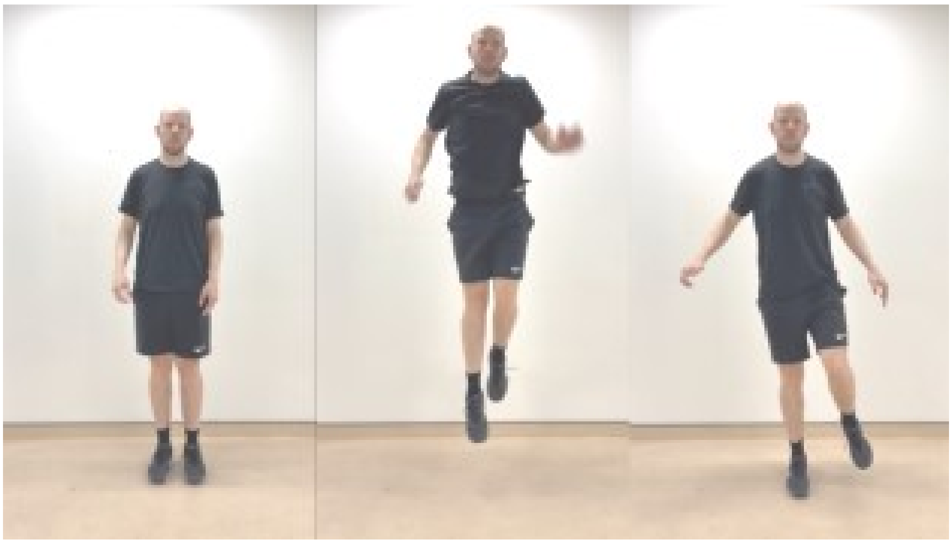

Track: Landing Technique

Sets: 2

Repetitions: 6

Duration: 2½-3 minutes

From a standing position, participants are instructed to perform a vertical jump and land on one leg. While in the air, a fellow participant applies a small push (perturbation) from a random direction (e.g. a push to the left shoulder, and in the second jump as push to the right shoulder). Participants are instructed to land as quietly as possible while maintaining proper lower limb alignment in the frontal plane, ensuring that the knee remains over the toes, and to maintain balance for ~3 seconds.
