# Supplementary material for: No Alterations in ACL Injury Risk Factors in Preadolescent Elite Female Handball Players Following an Eight‐Week Targeted Training Intervention: A Randomised Controlled Trial
Source: Transl Sports Med. 2026 Jan 2;2026:2570210. doi: 10.1155/tsm2/2570210 (PMC12782339; doi:10.1155/tsm2/2570210)
Supplement: Supplementary file 4 — Supporting Information 4 Supporting Information 4: Un‐normalised EMG and strength data. [file TSM2-2026-2570210-s001.pdf]

Supplementary Information 4: Unnormalised EMG and strength data

This supplementary file provides additional data on the unnormalised ST pre-activation EMG and maximal isometric hip external rotation strength presented in the main manuscript entitled *"No alterations in ACL injury risk factors in preadolescent elite female handball players following an eight-week targeted training intervention: A randomised controlled trial."*

Table S4. Descriptive data (mean ± SD) for the normalised and unnormalised ST pre-activation EMG and maximal isometric hip external rotation strength for the ACL-IPP and CON groups, respectively.

		ACL-IPP Gr.			CON Gr.	
	**Baseline**	**8 weeks**	**16 weeks**	**Baseline**	**8 weeks**	**16 weeks**
Bodymass (kg)	52.7±9.8	53.9±9.8	54.5±10.0	54.3±7.7	55.8±8.2	56.0±8.6
Hip Ext.Rot MVC (N)	116±21	115±20	122±19	109±15	113±14	114±18
Hip Ext.Rot MVC (N/kg BW)	2.2±0.3	2.2 ± 0.3	2.3 ± 0.4	2.0 ± 0.3	2.0 ± 0.3	2.1 ± 0.3
ST MVC EMG (mV)	692±285	607±191	546±187	827±215	782±284	767±326
Sidecut ST Pre-Activation (mV)	229±89	218±96	204±93	242±123	214±116	188±99
Sidecut ST Pre-Activation (% of max EMG)	28.7±10.6	30.2±14.9	28.2±13.3	35.0±13.3	34.8±15.3	34.2±14.2

N: Newtons; mV: millivolts; BW: Bodyweight; MVC: Maximum voluntary contraction.
